# Monitor unit verification for Varian TrueBeam VMAT plans using Monte Carlo calculations and phase space data

**DOI:** 10.1002/acm2.14063

**Published:** 2023-07-19

**Authors:** Ankit Pant, Narges Miri, Stephen Bhagroo, Joshua A. Mathews, Daryl P. Nazareth

**Affiliations:** ^1^ Department of Radiation Medicine Roswell Park Comprehensive Cancer Center Buffalo New York USA; ^2^ Medical Physics Program University at Buffalo (SUNY) Buffalo New York USA; ^3^ Department of Radiation Oncology Huntsman Cancer Institute Salt Lake City Utah USA; ^4^ Tacoma Valley Radiation Oncology Centers Tacoma Washington USA

**Keywords:** independent calculation verification, monitor units, Monte Carlo calculation, phase space, RapidArc, secondary check, VMAT

## Abstract

To use the open‐source Monte Carlo (MC) software calculations for TPS monitor unit verification of VMAT plans, delivered with the Varian TrueBeam linear accelerator, and compare the results with a commercial software product, following the guidelines set in AAPM Task Group 219. The TrueBeam is modeled in EGSnrc using the Varian‐provided phase‐space files. Thirteen VMAT TrueBeam treatment plans representing various anatomical regions were evaluated, comprising 37 treatment arcs. VMAT plans simulations were performed on a computing cluster, using 10^7^–10^9^ particle histories per arc. Point dose differences at five reference points per arc were compared between Eclipse, MC, and the commercial software, MUCheck. MC simulation with 5 × 10^7^ histories per arc offered good agreement with Eclipse and a reasonable average calculation time of 9–18 min per full plan. The average absolute difference was 3.0%, with only 22% of all points exceeding the 5% action limit. In contrast, the MUCheck average absolute difference was 8.4%, with 60% of points exceeding the 5% dose difference. Lung plans were particularly problematic for MUCheck, with an average absolute difference of approximately 16%. Our EGSnrc‐based MC framework can be used for the MU verification of VMAT plans calculated for the Varian TrueBeam; furthermore, our phase space approach can be adapted to other treatment devices by using appropriate phase space files. The use of 5 × 10^7^ histories consistently satisfied the 5% action limit across all plan types for the majority of points, performing significantly better than a commercial MU verification system, MUCheck. As faster processors and cloud computing facilities become even more widely available, this approach can be readily implemented in clinical settings.

## 1 INTRODUCTION

Radiation therapy has been subject to many advances in treatment planning and delivery over the years, at the cost of added complexity with each improvement. In such complex systems, such as IMRT/VMAT treatment, there is the potential for errors in software, hardware, networking, or mechanical components to result in inaccurate or even unsafe treatment delivery. One of the major motivations for the initial physics check (IPC) is to minimize the possibility of any failures in these subsystems. A proper IPC helps ensure safe and accurate treatment delivery while avoiding potentially serious misadministrations.^1^, ^2^

Secondary MU calculations have been used for MU verification (MUV) since the 1950s to confirm the dose calculations of a treatment planning system (TPS).^1^, ^3^ Although the TPS's accuracy and safety are generally validated by a comprehensive set of acceptance and commissioning tests, there are scenarios where these tests are not possible or do not apply. Some clinics may not have the resources available for performing a complete set of tests. Even when feasible, commissioning tests for a TPS cannot encompass the full range of clinical scenarios that may be encountered. This deficiency will be even more prominent in the future as planning systems evolve with newer functionalities and complexities. This is highlighted by the IROC‐Houston report, which indicates that most errors in IMRT delivery involved TPS dose calculation errors.^1^, ^2^, ^4^ Therefore, secondary MU calculations can serve as a valuable tool in identifying dose computation errors during the IPC.

While hand calculations have been used in the past to verify simple 3D fields, given the ever‐increasing complexity of planning and delivery associated with IMRT/VMAT treatments, these methods are no longer reliable.^1^, ^2^, ^4^ An important criterion for MUV set by TG‐114 and TG‐219 is the need for independence from the primary calculation; that is, MUV methods should use algorithms or software different from those of the TPS. This will avoid the possibility of systematic calculation errors.^1^, ^2^ To this end, various commercial software systems are available for performing independent calculations, with RadCalc (Lifeline Software Inc., Tyler, TX) being reported as the most commonly‐used software, along with Diamond (PTW, Freiburg GmbH, Germany), IMSure (Standard Imaging Inc., Middleton, WI), and MUCheck (Oncology Data Systems, Inc., Oklahoma City, OK).^1^

Secondary calculations typically employ simpler dose calculation algorithms than those used in primary calculations, with the modified Clarkson integration being the most common method (e.g., RadCalc).^1^, ^2^, ^3^ However, this can result in large discrepancies between primary and secondary results, especially in regions of tissue heterogeneity or large dose gradients. The MUV software attempt to correct for heterogeneities by employing the simple equivalent‐depth approach, which reduces their accuracy in many situations.^1^, ^3^, ^4^, ^5^ Furthermore, as these MUV software use single‐point calculations, they fail to account for the effects of nearby heterogeneities not in the direct beam path.^4^, ^6^ In addition, small field sizes, which are typically used in many VMAT/SBRT plans, present calculation challenges related to the lack of electronic equilibrium, and are not adequately modeled by simple MUV algorithms.^3^, ^5^

Our institute uses MUCheck (version 10.2.0) with the VMAT module (version 9.5.0) to perform independent MUV calculations for VMAT plans. As indicated above, the MUCheck algorithm employs a simple method where dose is calculated to a single point. Tissue density and type interfaces are only taken into account along the line joining the source to the point of interest (i.e., effective depth calculation); nearby heterogeneities may not be considered during calculation. Dose contributions from each control point aperture is calculated using a modified Clarkson integration. MUCheck uses HU‐to‐electron density CT calibration data along with patient‐specific CT and RT DICOM files for dose/MU calculation.^4^, ^6^

Since the dose calculation algorithms used in a TPS are more sophisticated than those used in MUV software, it has been suggested instead to use algorithms of similar accuracy to avoid large discrepancies.^2^, ^3^, ^4^, ^5^ Our institute's TPS is Eclipse version 15.6 (Varian Medical System, Palo Alto, CA), with Acuros XB (AXB) as the dose calculation algorithm. Acuros XB is more accurate than superposition/convolution algorithms (e.g., AAA), particularly in heterogeneous regions.^7^, ^8^, ^9^ AXB is a deterministic grid‐based Boltzmann solver (GBBS) that explicitly solves the linear Boltzmann transport equation (LBTE), which describes the macroscopic behavior of radiation particles (photons, electrons, etc.) propagating through and interacting with medium. Monte Carlo (MC) methods represent another approach to solving the LBTE, by using random sampling techniques. MC and AXB have been found to be in good agreement with one another in a large sample of clinical situations, and, under suitable calculation conditions, AXB and MC results should be convergent.^10^, ^11^

MC‐based secondary calculations are also useful because they can produce a complete 3D dose distribution matrix, allowing for more sophisticated analysis of a complex VMAT plan (e.g., gamma analysis).^1^, ^4^ Their disadvantage has historically been their long calculation times and large computing resource requirements.^4^, ^10^ However, with the availability of more powerful computers, parallel computing approaches, and cloud computing solutions, MC‐based calculation methods can be computationally efficient and feasible in clinical settings.^4^, ^12^

Bhagroo et al.^4^ demonstrated that the MC approach could be applied to Varian Trilogy using the EGSnrc MC package. Varian Medical Systems released a successor linac platform to Trilogy, TrueBeam, that can feature the newer multi‐leaf collimator and flattening‐filter‐free (FFF) clinical beams for 6 and 10 MV energies. Unlike the Trilogy, specifications of the TrueBeam's treatment head components are proprietary, so a full MC simulation of the accelerator head is not possible. Instead, Varian provides precalculated energy‐specific phase‐space files located at the horizontal plane above the secondary collimators, which can be treated as a set of photon sources for MC simulation purposes.^13^ This approach does involve some known limitations, such as the statistical uncertainty associated with latent phase space variance and the inability to adapt the virtual model to reflect the user's linac precisely.^14^

While the TrueBeam adds various features that enables more sophisticated beam delivery, these features can also add more complexities to dose calculation that affect the calculation accuracy. At our institution, the TrueBeam is used routinely for complex SRS/SBRT cases like lung treatments. Such scenarios can involve FFF beam, heterogenous media, high‐dose gradient, and small fields, all which can lead to large disagreements between simple and more sophisticated algorithms. Large disagreements (>20%) have been observed between MUCheck and our Eclipse TPS, particularly for lung cases.^7^ The research literature shows that MC calculations, however, can perform favorably in such complex scenarios compared to simpler commercial software.^1^, ^4^, ^12^, ^15^ Based on these ideas, we aim to develop a phase‐space based MC TrueBeam model with HD‐MLCs for use in radiotherapy simulations as a fast and accurate secondary MU check system, employing parallel processing. Many of TrueBeam's newer features (e.g., FFF beam profiles) were used in this study for a range of VMAT cases.

## 2 MATERIALS AND METHODS

### 2.1 Initial setup

A virtual linear accelerator is typically modeled using the EGSnrc module, BEAMnrc. However, as indicated above, a TrueBeam simulation requires the use of phase‐space files provided by Varian. These phase‐space files are specific to each beam energy and contain data for ∼5 × 10^7^ particles. In order to decrease statistical variance as a result of the phase‐space simulation, this study used a concatenated phase‐space approach, in which five phase‐spaces files were aggregated into a single file with data for ∼2.5 × 10^8^ particles.

**FIGURE 1:**
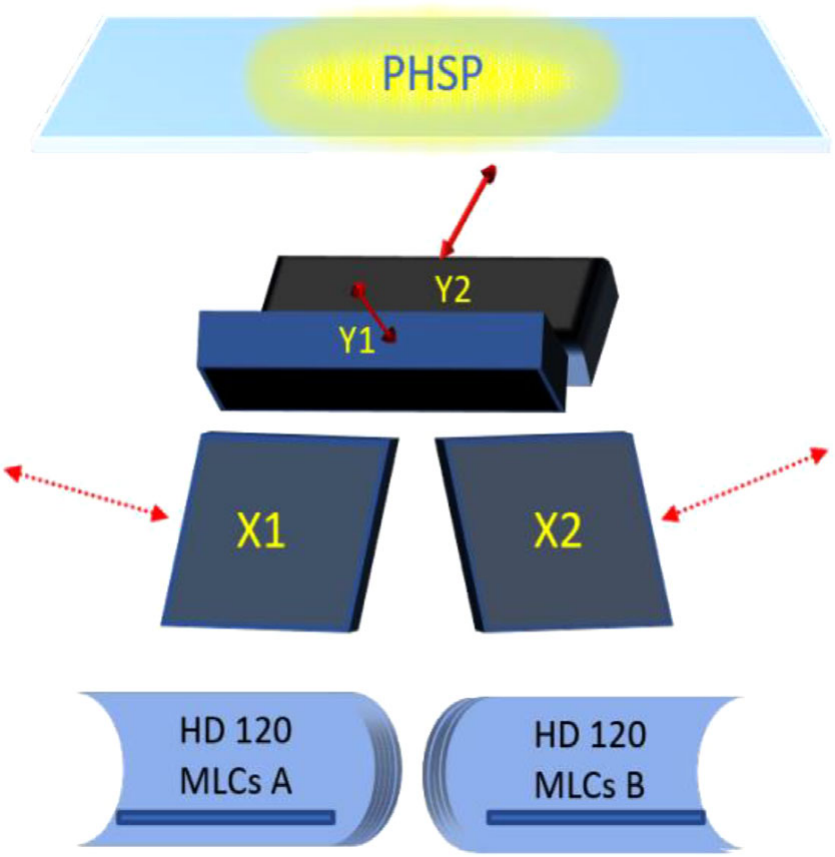
An illustration of accelerator components showing the location of phase space source plane along with components.

The accelerator can then be modeled with a BEAMnrc script by specifying downstream components (e.g., jaws and MLCs), as shown in Figure 1. The TrueBeam model used in this work features high‐definition multi‐leaf collimators (HD MLC). The modeling of these components involved the use of freely available Varian MC support documentation containing various specifications for the physical dimensions of HD MLCs. These specifications (leaf thicknesses, widths, screw hole size, groove length, etc.) were coded into the TrueBeam accelerator model using syntax specific to BEAMnrc's HD MLC module. A more in‐depth treatment of HD‐MLC and TrueBeam modeling is provided in Bhagroo.^16^

Another EGSnrc module, DOSXYZnrc, can then be used to model dose deposition, given some linac parameter input file and a voxelated phantom model. The simulation output is a 3D dose matrix file, where each voxel value is provided in the unit of Gy/particle. Converting these values to Gy is a standard process, which requires a calibration factor. The calibration factor can be determined by running a static plan under reference conditions (10 × 10 cm^2^ field, 100 cm SSD with a 40 × 40 × 40 cm^3^ water block phantom) in MC and Eclipse. Under these reference conditions, *F_Cal_* can be obtained from ratio between the Eclipse and MC dose at *d* = 10 cm on the central axis, as shown in Eqn. (1):

(1) $$F_{Cal}\;\left[\mathrm{particle}/\mathrm{MU}\right]=\frac{D_{Eclipse}\;\left[cGy\right]\;/\;MU_{Eclipse}\;\left[\mathrm{MU}\right]\;}{D_{MC}\;\left[\mathrm{Gy}/\mathrm{particle}\right]}\;$$

This study follows the calibration process of Bhagroo et al.^4^ and recommendations from TG‐157 and applies a calibration factor unique to each beam energy used in the evaluations: 6X, 6X‐FFF, 10X, and 10X‐FFF.^4^, ^17^

### 2.2 Monte Carlo calculations

**FIGURE 2:**
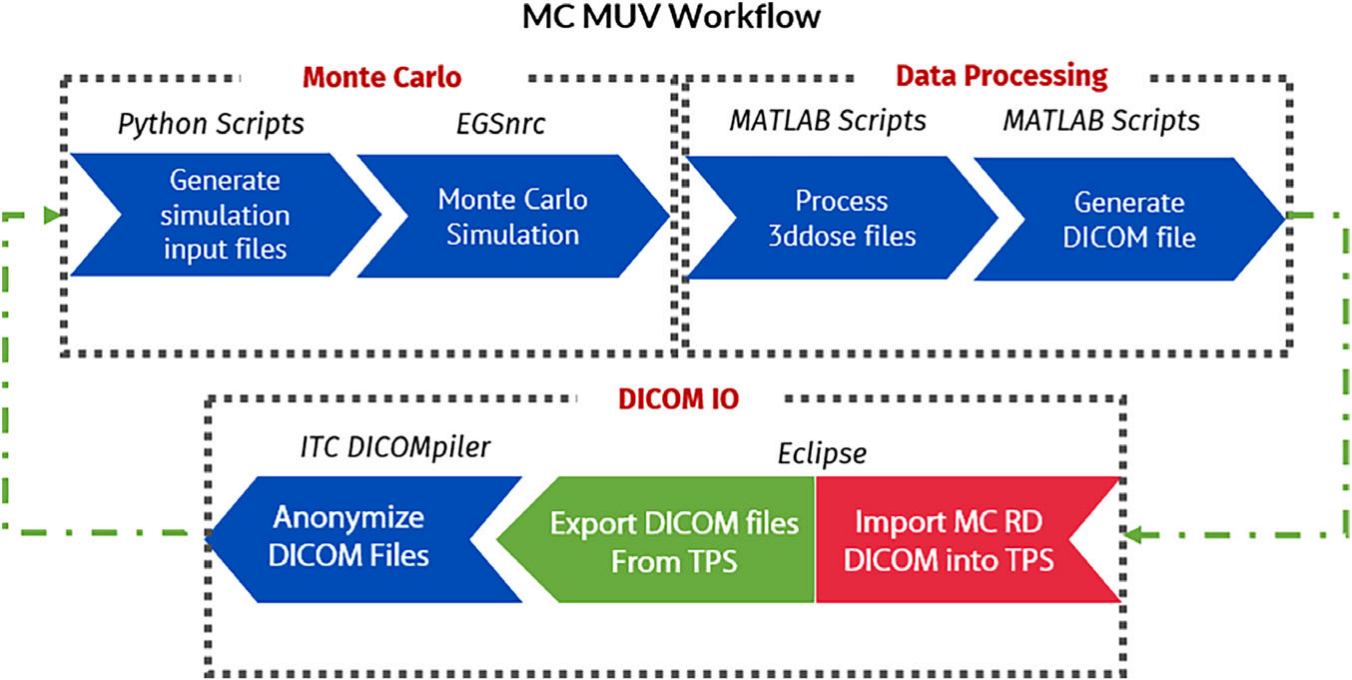
A flowchart showing the workflow of MC‐MUV process. Sub‐processes are shown in dashed borders. Software used throughout the steps are shown in italicized.

MC MUV is a three‐step process, shown in Figure 2. The DICOM IO (Input & Output) subprocess is the start and end of the workflow. Initially, the patient DICOM files (CT Image, RT Dose, Plan, and Structure files), are exported from the TPS and anonymized using ITC DICOMpiler.

Next, the RT Dose files are processed using in‐house script to create VMAT plan parameter files for BEAMnrc and DOSXYZnrc. Additionally, voxelated phantoms are created for DOSXYZnrc using the DICOM files. The phantom is defined using four different materials with a HU number interval (from the CT DICOM files) mapped to a density interval for a particular material (see Table 1). Our in‐house phantom generator then assigns each voxel in the virtual phantom a material value with a certain density. The simulation is performed after generating all the prerequisite files. Finally, the Data Processing subprocess verifies and exports the output into a new DICOM file for TPS import.

**TABLE 1:** Phantom material/density assignment based on Hounsfield Unit (HU).

Material	HU min	HU max	Density min (g/cm^3^)	Density max (g/cm^3^)
Air	−1050	−950	0.001	0.044
Lung	−950	−700	0.044	0.302
Tissue	−700	125	0.302	1.101
Bone	125	2000	1.101	2.088

The input generation processes, subsequent MC simulations, and data processing are performed on a distributed‐computing facility using file transfer and secure shell clients.^18^ The simulations are parallelized using an in‐house Linux shell script, by taking each DOSXYZnrc input file and creating multiple identical files. For example, a single job with 10^7^ histories can be parallelized into 100 smaller jobs, each with 10^5^ histories. Once each smaller job is completed, they will generate binary files called pardose files, containing various information about the simulation (e.g., dose deposition). The pardose files from all small jobs will be combined to obtain a single 3ddose file for the original job, which is imported into MATLAB (MathWorks Inc., Natick, MA).^19^ The calibration factor, *F*_Cal_, and monitor units for each VMAT arc are applied, and a new DICOM RT Dose file is created for importing into Eclipse.

### 2.3 Evaluations

The method was evaluated retrospectively on 13 patient plans, including 2 abdominal, 2 brain, 2 head and neck, 4 lung, and 3 prostate cases (see Table 2). Each plan included 2−4 VMAT arcs, totaling 37 arcs. All the plans were obtained from our institution's TPS, Eclipse, and the plans were calculated using version 15.6.05 of Photon Optimizer and Acuros XB. In order to investigate the effect of varying the number of histories, each MC arc simulation employed five different numbers of histories: 10^7^, 2 × 10^7^, 5 × 10^7^, 10^8^, and 10^9^.

**TABLE 2:** Plan information used in this study. Brain 1 has multiple PTV and Brain 2 has different number of control points for arcs.

Plan	Energy	Arcs	Control points per arc	Dose grid [mm]
Abdominal 1	6FFF	3	114	1.25
Abdominal 2	10FFF	3	114	1.25
Brain 1	6FFF	3	98	1.25
Brain 2	6X	4	178 × 2 98 × 2	2.0
Head & Neck 1	10FFF	3	178	1.25
Head & Neck 2	6FFFF	3	178	1.25
Lung 1	6FFF	3	114	1.25
Lung 2	6FFF	3	114	1.25
Lung 3	6FFF	3	98	1.25
Lung 4	6FFF	3	114	1.25
Prostate 1	10X	2	178	2.0
Prostate 2	10X	2	178	2.0
Prostate 3	6X	2	178	2.0

A common parallelization scheme was employed across all plans. The simulations were parallelized on multiple Intel Xeon Gold 6130 processors (16 physical cores, 32 logical CPUs). With most plans having three arcs, individual arcs were run using 320 jobs, for a total of 960 jobs per plan; this was to ensure all jobs for each arc completed in similar time while maximally utilizing the 1000 job limit of the computer cluster. Each individual job was assigned to run on a single CPU, that is, 960 jobs run on 960 CPUs.

Five 3D reference points were specified within the PTV for each plan for absolute dose calculation comparison. These points included the PTV centroid and four others, 0.5–1 cm away in the superior/inferior or lateral directions, depending on the PTV size. The percentage dose differences between Eclipse and MC were compared with those between Eclipse and MU Check. The time required for the MC calculations was also evaluated. For MUCheck calculations, the RT (dose, plan, and structure), and CT DICOM files were exported from the TPS. MUCheck determined the dose difference between the RT Dose value and its own calculation.

## 3 RESULTS

### 3.1 Point dose differences

**FIGURE 3:**
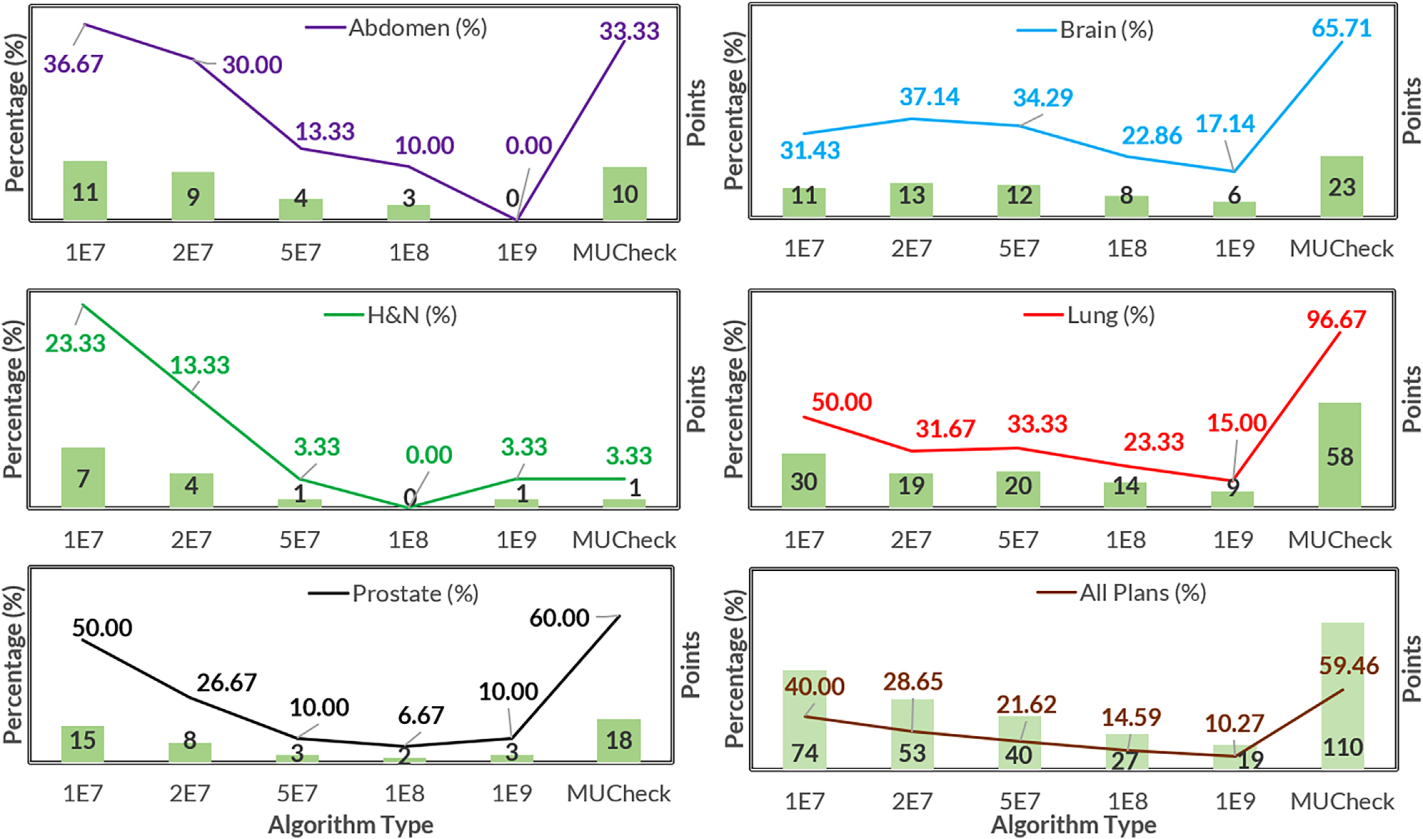
Percentage of reference point dose differences greater than 5% for the various cases and methodologies. MUCheck differences tend to be larger, except for head & neck cases.

Figure 3 shows the number/percentage of points with dose differences between TPS and secondary MU that exceed the 5% action limit for various secondary MU calculation (five MC and one MUCheck) methodologies across various treatment sites. For MUCheck, approximately 60% of all points exceeded the 5% threshold. For MC, this decreased from 40% to 10% with increasing histories. MUCheck exhibited particularly high differences for lung (97%), brain (66%), and prostate (60%) cases. The corresponding numbers for MC with 10^8^ histories were 23%, 23%, and 7% and 15%, 17%, and 10% for 10^9^ histories.

**FIGURE 4:**
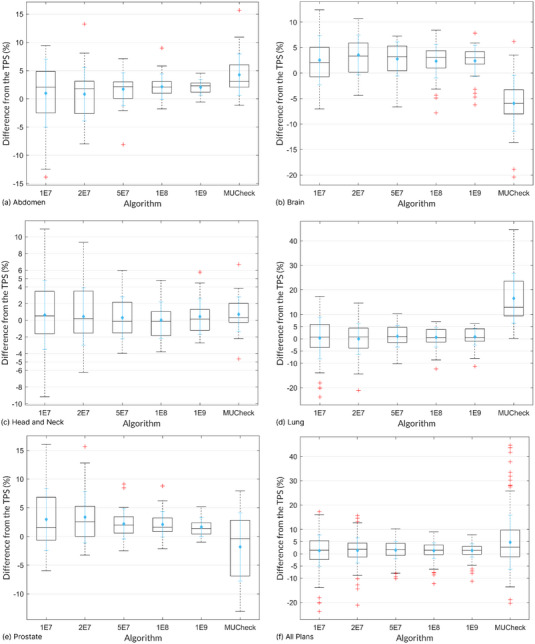
Reference point dose differences between TPS and MC histories and MUCheck methodologies across various sites. Boxes show 25th (Q_1_), 50th (Q_2_), and 75th (Q_3_) percentiles; whiskers show maxima and minima within 1.5 interquartile range (Q_3_‐Q_1_); + symbols show outliers. Average and standard deviation of reference point dose differences are shown as blue diamond and line respectively.

Figure 4 shows the comparison of dose difference between TPS and each secondary MU calculation methodologies across the various treatment sites. These box‐plot distributions, indicate each median, 25th percentile, 75th percentile, maximum and minimum within 1.5 interquartile range (IQR), and outlier; additional distribution statistics was provided by average and standard deviation of the dose difference. The spread of differences decreased for every treatment site as the number of MC histories increased. For 10^8^ and 10^9^ histories, the 25th to 75th percentiles all lie within the 5% action limit; the average was always within 5% action limit for all MC calculations. In comparison, the MUCheck 25th—75th percentiles all have values outside the 5% limit, except for the head and neck cases; for Brain and Lung, the average also lay outside the 5% limit. The largest outlier for the MC values decreased from 25% to 11% as histories increased. In comparison, the largest MUCheck outlier was 45%. Note that some cases with higher histories see increases in outliers. This may be due to increasing histories resulting in smaller IQR (smaller whiskers) and some reference points’ dose difference not having increased agreement even with higher histories. This leads to certain dose differences being indicated as outliers, which may not have been the case with lower histories.

**FIGURE 5:**
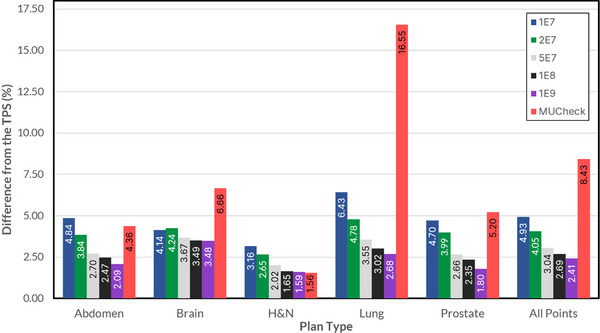
Average absolute reference point dose difference across various cases. MUCheck differences are particularly large for lung cases.

Figure 5 shows the average absolute reference point dose difference for each methodology. For each treatment site, all reference points’ dose differences were collected and the absolute of all those values were averaged. Increasing the histories tends to decrease the average absolute difference for MC values. The average absolute difference for brain, lung, and prostate cases was greater than 5% for MUCheck, but only greater than 5% for MC lung cases with 10^7^ histories.

### 3.2 Computing performance

Each MC secondary MU calculation consists of both overhead and simulation tasks. The overhead tasks include creating the patient phantom CT and plan parameter files, as described in Methods. Recall that each simulation is parallelized into 320 concurrent jobs. Employing 1000 concurrent jobs was investigated, but this did not have a significant effect (see Discussion). The values reported here are the times required per case and not per arc.

**FIGURE 6:**
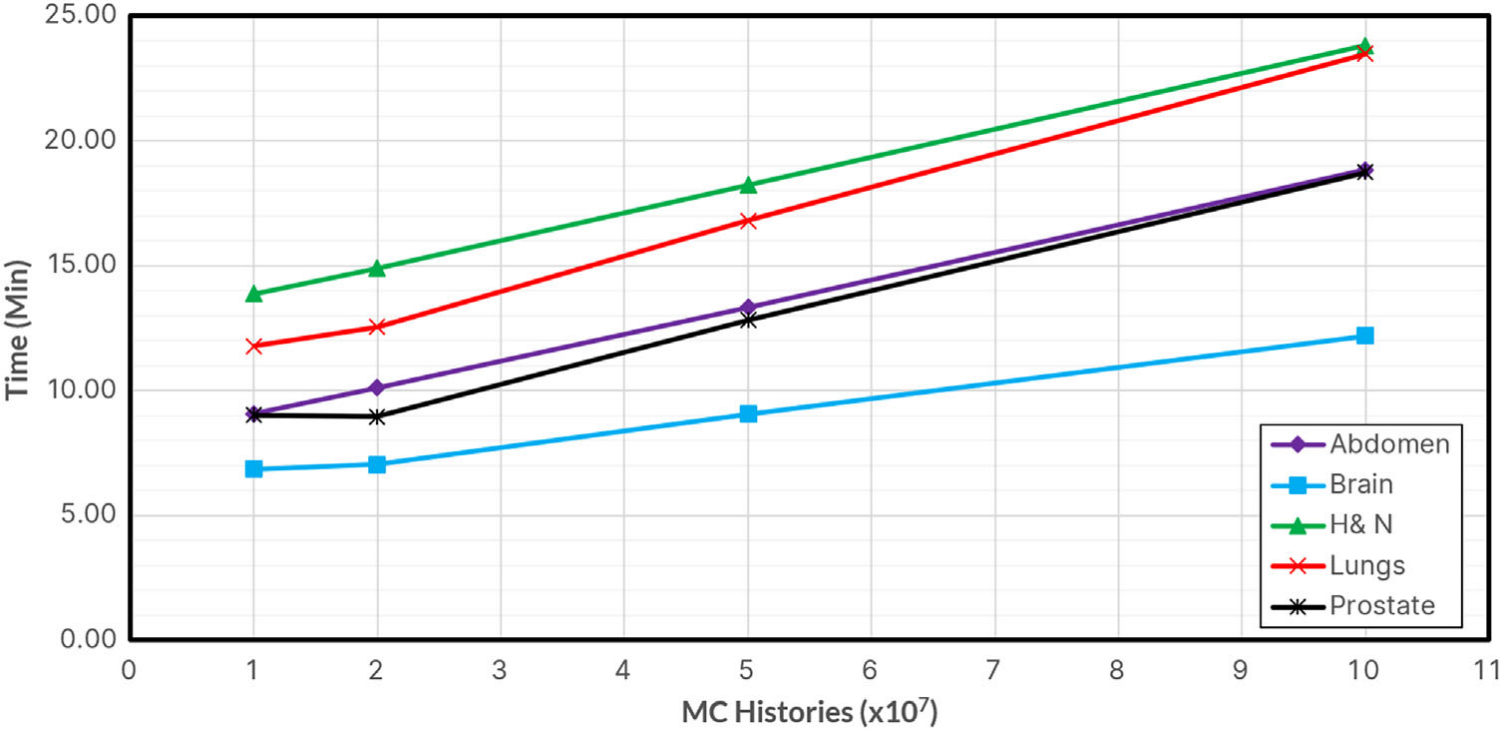
Total average time required for MC calculations, including overhead, for each plan type across various histories.

**FIGURE 7:**
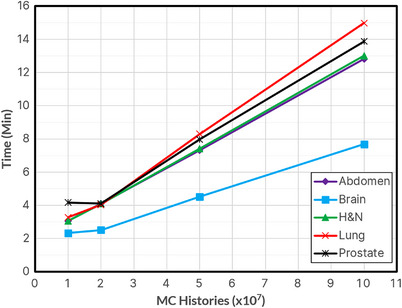
Average MC simulation time for each plan type across various histories.

**FIGURE 8:**
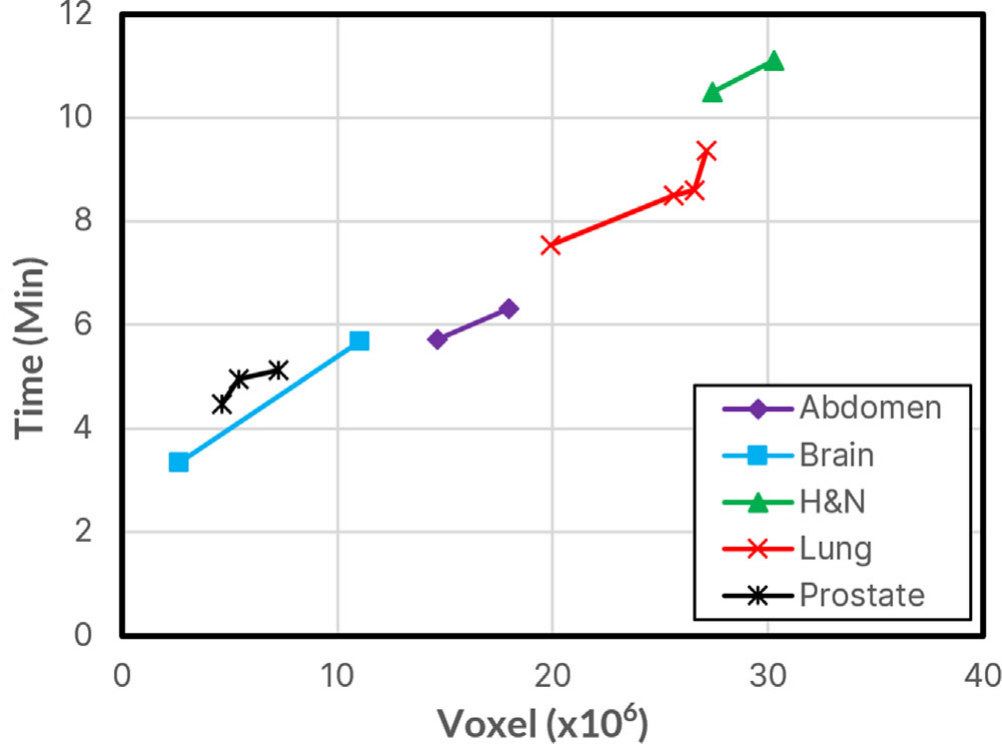
Overhead time required for MC calculations as size of the dose matrix varies. Head & Neck and Lung cases have the largest sizes.

Figure 6 shows the average time required for the entire MC calculation, including overhead time, for the various cases and 10^7^ through 10^8^ histories. Head & Neck and Lung plans required the longest total time (24 min maximum), while Brain plans were the shortest (12 min maximum). 10^9^ histories simulation results are not displayed due to their large values, averaging around 45 min to 2.5 h. Figure 7 shows the average time required per simulation. Lung and Prostate plans required the longest simulation times, while brain simulation times were still the shortest. Figure 8 shows the overhead time required (3–11 min), as the dose matrix size increases, with the number of voxels for each plan type shown.

## 4 DISCUSSION

The results obtained in this work suggest that MC‐based monitor unit verification can provide reliable and accurate secondary calculations for TrueBeam VMAT plans across varying anatomy types and beam energies, including FFF beams.

For all the plans evaluated in this study, the average (absolute) difference from the TPS was less than 5% for all the various MC histories calculations. For histories of 5 × 10^7^ and above, the MC calculations had ∼3% or below average differences, and more than 80% of the 185 reference points (across all arcs and plans) had dose differences within ± 5% of the TPS, which is considered the action limit. This compares well with a commercial secondary MU calculation software package, MUCheck, which had differences within 5% of the TPS for only 41% of reference points. It should be noted, as the focus of this study was to evaluate our MC MU calculation and MUCheck, 3D dose assessment was not examined here as MUCheck only supports single point evaluation. However, as MC calculations produce 3D dose distribution, it is possible to compare with the TPS calculated 3D dose distribution using more sophisticated method, for example, gamma analysis. Future work will involve such type of assessment and we are currently developing software to perform 3D gamma analysis between TPS and MC 3D dose distribution.

It is well known that the accuracy of MC calculations increases with increasing particles or histories, and our results are consistent with this trend. While the median and the average difference between all histories were similar, the average absolute difference was greatest for 10^7^ histories, and smallest for 10^9^ histories. The median and average differences include positive and negative values, which tend to cancel, and, therefore, will be small, regardless of the number of histories. For 10^7^ histories, 40% of points exceeded the action limit, comparable to the results of MUCheck. Therefore, we recommend at least 5 × 10^7^ histories be used for this application. This is slightly higher than the value of 10^7^ determined by Bhagroo et al.^4^; however, those comparisons were made with Eclipse's AAA dose calculation system, which is less accurate than AXB. In addition, our current evaluations involve many SBRT/SRS plans with much smaller field sizes.

There were a few instances where some reference points saw increases in dose differences with 10^9^ histories; such instances may highlight the limit of agreement achievable between EGSnrc and AXB, which is subjected to resource constraints typical of clinical treatment planning.^20^, ^21^

The MC simulation time was related to the number of histories, ranging from 2−4 min to 45−120 min for 10^7^ and 10^9^ histories, respectively. The time was also dependent on the dose grid size and extent of inhomogeneities in the CT image, with the lung requiring the most time. The overhead time included phantom creation, parameter file creation, pardose combination, and 3ddose data processing. This time depended on the number of dose voxels but was independent of the number of histories. The total time was the sum of simulation and overhead times, ranging from 9−18 min for 5 × 10^7^ and 12−24 min for 10^8^. However, more efficient parallelization during the phantom‐generation process may reduce this time further. Due to clinical considerations, it is apparent that simulations of 10^9^ histories, which may take more than 1 h to complete, are not feasible in practice. As indicated above, 10^7^ histories do not provide the necessary accuracy for this application. Therefore, we recommend a range of 5 × 10^7^ to 10^8^ histories for secondary MU calculations. In comparison, when MUCheck is used in our clinic, it requires 7−10 min to verify the dose calculation at a single 3D point for all arcs of a typical VMAT plan.

Both the overhead and simulation tasks were performed on a distributed computer cluster. The results were obtained by parallelizing each simulation into 320 jobs. However, we also investigated the use of 1000 jobs per simulation. Although this reduced simulation time, it also increased overhead time due to the additional jobs created and processed. The total time, therefore, increased in many cases of 5 × 10^7^ histories and some cases of 10^8^ histories. We, therefore, recommend a parallelization level of about 320 concurrent jobs.

Although head and neck cases can present with heterogeneities, simple radiological depth corrections used in MUV software has been known to provide reasonable accuracy for H&N cases and as such MUCheck performs relatively well for such cases.^1^ However, the VMAT lung cases proved particularly challenging for the MUCheck software due to their large inhomogeneities involving possibly multiple interfaces between soft tissue and lung. Of the 60 reference points evaluated, 58 resulted in dose difference with the TPS of more than 5%. One approach to dealing with this inaccuracy, which had been previously adopted in our clinic, is to create a new treatment course in Eclipse, specify water‐equivalent HUs within the CT body contour, and recalculate the dose using fixed MUs. Although this procedure improves the agreement between MUCheck and Eclipse, it increases the time required for an initial physics chart check. It also potentially reduces the value of the secondary MU verification process since the actual clinical dose calculation is not being verified.

While MC simulations can provide robust and accurate MU verifications, the required computational resources are a consideration. Although not every center has dedicated computing facilities used for this study, cloud computing is widely available and is becoming increasingly inexpensive. In addition, new approaches to reducing MC simulation time by applying machine learning algorithms are being actively investigated.^22^, ^23^

## 5 CONCLUSION

The use of MC calculations, based on the EGSnrc/BEAMnrc framework, has been evaluated for MU verification of Varian TrueBeam VMAT plans. The application of this method to SBRT/SRS plans with FFF beams that employ HD MLCs, using a phase‐space calculation approach is novel and has provided results significantly more accurate than the commercial clinical software, MUCheck. Although our phase‐space approach here focused on the Varian TrueBeam, this method should be applicable to other treatment machines where phase‐space file(s) and appropriate downstream modeling (i.e., secondary jaws and MLC) are available.

With the recommended 5 × 10^7^ histories, the average absolute dose difference between MC simulations and Eclipse Acuros XB was 3%, while 78% of reference points were within the 5% action level. In comparison, the average absolute difference for MUCheck was 8%, with only 41% of reference points lying within 5% of AXB. By optimizing the parallelization of the MC computations, the MU verifications may be performed in a clinically‐feasible timeframe while providing an accurate secondary calculation method across varying treatment sites and delivery complexities.

## AUTHOR CONTRIBUTIONS

All authors contributed substantially to the conception and design of work, data analysis and interpretation of data, editing of the manuscript, and final approval of the version to be published. Ankit Pant drafted the manuscript and did data acquisition.

## CONFLICT OF INTEREST STATEMENT

The authors declare no conflicts of interest.
